# Surgery, with or without tamoxifen, *vs* tamoxifen alone for older women with operable breast cancer: Cochrane review

**DOI:** 10.1038/sj.bjc.6603600

**Published:** 2007-02-06

**Authors:** D Hind, L Wyld, M W Reed

**Affiliations:** 1 School of Health and Related Research (ScHARR), University of Sheffield, Sheffield, UK; 2 Department of Surgical Oncology, University of Sheffield, Academic Surgical Oncology Unit, Sheffield, UK

**Keywords:** aged, breast neoplasms, surgery, randomised controlled trials, tamoxifen

## Abstract

The published literature comparing surgery, with or without adjuvant endocrine therapy, with endocrine therapy alone in older women with operable breast cancer was systematically reviewed.The design used is Cochrane review. Randomised controlled trials retrieved from the Cochrane Breast Cancer Group Specialised Register on 29 June 2005. Eligible studies recruited women aged 70 years or over with operable breast cancer, fit for surgery under general anaesthia. The studies compared surgery (either mastectomy or wide local excision, with or without endocrine therapy) to endocrine therapy alone. Primary outcomes were overall survival (OS) and progression-free survival (PFS). Double data extraction and quality assessment were undertaken. Seven eligible trials were identified of which six had published time-to-event data. The quality of the allocation concealment was adequate in three studies and unclear in the remainder. In each case the endocrine therapy used was tamoxifen. When surgery alone was compared to endocrine therapy alone, there was no significant difference in OS (hazard ratio (HR) 0.98, 95% confidence interval (CI) 0.74–1.30, *P*=0.9), but a significant difference in PFS (HR 0.55, 95% CI 0.39–0.77, *P*=0.0006). When surgery with adjuvant endocrine therapy was compared to endocrine therapy alone, there was no significant difference in OS (HR 0.86, 95% CI 0.73–1.00, *P*=0.06), but a significant difference in PFS (HR 0.65 (95% CI 0.53–0.81, *P*=0.0001) for surgery plus endocrine therapy *vs* primary endocrine. The regimens have different side effect profiles with one study suggesting increased psychosocial morbidity at 3 months in the surgical arm, which resolves by 2 years. Primary endocrine therapy with tamoxifen is associated with inferior local disease control but non-inferior survival to surgery for breast cancer in older women. Trials are needed to evaluate appropriate selection criteria for its use in terms of patient co-morbidity and quality of life. Trials are needed to evaluate the clinical effectiveness of aromatase inhibitors as primary therapy for this population.

The standard treatment for early-stage breast cancer in women of all ages was surgery until the late 1970s (Kesseler and Seton, 1978). Primary endocrine therapy was first described in the early 1980s as an alternative for older women (Preece *et al*, 1982). Treatment involved the sole use of tamoxifen, an oestrogen-receptor antagonist, without surgery, radiotherapy or chemotherapy. Tamoxifen is effective in around 80% of women with moderately or strongly ER-positive tumours (Gaskell *et al*, 1992), but the duration of local disease control is shorter than with surgery, and some women require either alternate endocrine therapies or surgery at a later date (Kenny *et al*, 1998).

In the UK, the trend towards treating women aged 70 and over with tamoxifen alone has been based on the premise that they are less likely to be fit for surgery because of co-morbidity (Satariano and Ragland, 1994). However, both mastectomy and wide local excision have low mortality rates (Hunt *et al*, 1980; Wyld and Reed, 2003). Breast surgery-related morbidity, especially where axillary surgery is involved, is quite high and may impact on quality of life. Primary endocrine therapy is not a treatment option in the USA and is rarely used in Australia (Craft *et al*, 2000; Diab *et al*, 2000). In the UK, its use is widespread, with up to 42% of all women over 70 being treated in this way, regardless of whether co-morbidity is documented (Wyld *et al*, 2004).

To establish whether primary endocrine therapy is justifiable for women who are fit for surgery, we systematically reviewed the evidence from randomised trials comparing primary endocrine therapy to surgery, with or without adjuvant endocrine therapy, in the management of women aged 70 years or over with operable breast cancer.

## METHODS

Full details of the methods employed are published elsewhere (Hind *et al*, 2006). The Cochrane Breast Cancer Group Specialised Register was searched on 29 June 2005. Citations coded as ‘EARLY BREAST CANCER’, ‘ENDOCRINE THERAPY’, ‘PSYCHOSOCIAL’ or ‘SURGERY’ on the specialised register were retrieved with no date or language restrictions.

Only controlled trials with the following characteristics were included. Participants were women aged 70 years or over with clinically defined operable primary breast cancer: TNM classification T1-3 and T4b where there was only minor skin involvement, N0-1, mobile lymph nodes (Union Internationale Contre le Cancer, 1987). Studies had to compare either (1) surgery alone *vs* primary endocrine therapy; or (2) surgery plus adjuvant endocrine therapy *vs* primary endocrine therapy. Mastectomy could be with or without axillary clearance, and wide local excision could be with or without radiotherapy. Primary outcomes were overall survival (OS) and progression-free survival (PFS) (interval between start of treatment and need for second-line or palliative treatment, recurrence or death from any cause). Secondary outcomes were adverse effects (number of surgical complications or endocrine therapy related side effects), local disease control (interval between start of treatment and the development of local disease), distant metastasis-free interval (interval between start of treatment and the development of metastatic disease) and quality of life (however measured). Pre-specified subgroups included the type of surgery (mastectomy or wide local excision, with or without radiotherapy).

Two reviewers, LW and DH, independently assessed each potentially eligible trial for inclusion in the review with the results section masked. The same two reviewers independently reviewed each study according to its design and by how the study was conducted to assess any bias. The checklist for quality of randomised controlled trials included: concealment of the allocation sequence, generation of the allocation sequence, comparability between groups at the baseline and inclusion of all randomised participants in the analysis. For allocation concealment, trials were graded ‘A’ (adequate concealment), ‘B’ (adequacy of concealment unclear) or ‘C’ (clearly inadequate concealment).

The most complete data set feasible was assembled from the published literature. Where necessary, we sought additional information from the principal investigator of the trial concerned. Results of eligible studies were statistically synthesised if appropriate and possible. For time-to-event analyses, combined hazard ratios (HRs) and 95% confidence intervals (CIs) were estimated using formal methods for extracting summary statistics to perform meta-analyses of the published literature (Parmar *et al*, 1998). Heterogeneity between trial results was tested using the *χ*^2^ test and the *I*^2^ measurement (Higgins *et al*, 2003). Absolute risk reductions and numbers needed to treat were calculated using the Altman and Andersen (1999) method.

## RESULTS

**Figure 1:**
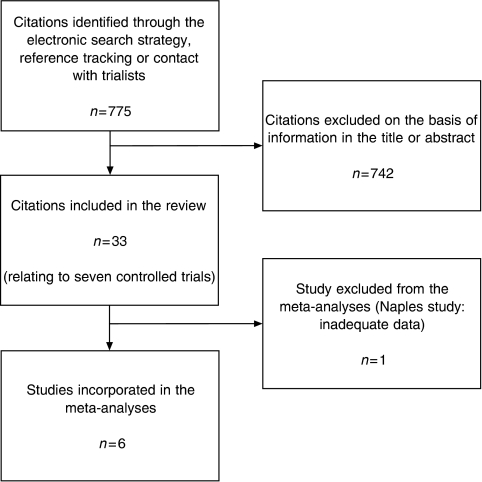
QUOROM flow diagram showing study selection process.

The search strategy retrieved 770 citations. Of these, 742 were excluded based on information in the title or abstract. The remaining 28 citations reported on the seven potentially eligible studies for the review. None of these studies was excluded. Five additional papers, all conference abstracts, relating to the same trials were identified through informal reference tracking and contact with authors. The study selection process is illustrated in Figure 1, in accordance with the QUOROM statement (Moher *et al*, 1999).

Three eligible trials addressing surgery *vs* primary tamoxifen therapy were identified (Table 1), all of which reported outcome data (Gazet *et al*, 1994; Kenny *et al*, 1998; Fentiman *et al*, 2003). None of the studies evaluated the oestrogen receptor status of the women they recruited. Four eligible trials addressing surgery plus endocrine therapy *vs* primary endocrine therapy were identified, of which three have reported outcome data (Willsher *et al*, 1997; Capasso *et al*, 2000; Mustacchi *et al*, 2003; Fennessey *et al*, 2004), although there is currently no data from one in a form that can be meta-analysed (Capasso *et al*, 2000). Not all trials identified provided adequate information on all outcomes. Only one of the studies evaluated oestrogen receptor status, recruiting only women with moderately or strongly oestrogen receptor-positive tumours (Willsher *et al*, 1997).

It was not possible to assess accurately the quality of all studies (including the quality of the randomisation process) owing to lack of information in the published articles. The quality of three trials was graded as A (Fentiman *et al*, 2003; Mustacchi *et al*, 2003; Fennessey *et al*, 2004) with the rest being graded as B (Gazet *et al*, 1994; Willsher *et al*, 1997; Kenny *et al*, 1998; Capasso *et al*, 2000). No potentially eligible study was excluded from the review. Proposed sensitivity analysis based on trial quality was not conducted because of the small number of trials identified. There was good agreement on study selection, quality assessment, and data extraction.

Results are presented in Table 2. Analysis of OS, based on three trials (495 women: Gazet *et al*, 1994; Kenny *et al*, 1998; Fentiman *et al*, 2003), showed no significant difference between interventions (HR 0.98, 95% CI 0.74–1.30, *P*=0.9). One trial (164 women: Fentiman *et al*, 2003) reported adequate summary data to show a significant difference in PFS, favouring surgery (HR 0.55, 95% CI 0.39–0.77, *P*=0.0006). In the surgery arm, 77% of women had died or progressed compared to 84% in the tamoxifen arm: an extra 7% of participants receiving surgery benefited from the treatment. For every 14 women treated, one death or disease progression would be prevented over 120 months.

Methodological issues (discussed below) prohibited either the meta-analysis of, or dissemination of results from the three trials, which reported data on local disease control or the distant metastasis-free interval. One trial (200 women: Gazet *et al*, 1994) reported adverse events. No patient discontinued primary tamoxifen treatment. Eight patients had a total of 10 side effects, including hot flushes, skin rash, vaginal discharge, indigestion, breast pain, and sleepiness. No trial reported quality of life data.

Results are presented in Table 3. Three trials (1076 women: Willsher *et al*, 1997; Mustacchi *et al*, 2003; Fennessey *et al*, 2004) reported data on OS which could be meta-analysed. There was a nonsignificant trend in favour of surgery plus endocrine therapy (HR 0.86, 95% CI 0.73–1.00, *P*=0.06). Only one trial (474 women: Mustacchi *et al*, 2003) reported adequate data on PFS to calculate a significant difference favouring surgery plus endocrine therapy (HR 0.65, 95% CI 0.53–0.81, *P*=0.0001). In the surgery arm, 59% of women died or progressed compared to 80% in the tamoxifen arm: an extra 21% of participants receiving surgery benefited from the treatment. For every five women treated, one death or disease progression would be prevented over 80 months.

Analysis of two trials (929 women: Mustacchi *et al*, 2003; Fennessey *et al*, 2004) showed a significant difference in local disease control in favour of surgery plus endocrine therapy (HR 0.28, 95% CI 0.23–0.35, *P*< 0.00001). There was significant heterogeneity between the two studies (*χ*^2^ 2.90, *P*<0.09, *I*^2^ 65.6%), which is discussed below, although each individually showed a statistically significant difference in treatment effect favouring the surgery arm. Data from one trial (Willsher *et al*, 1997) were not included in this analysis as reported results were immature compared to the other two studies. Adequate data were not available to evaluate the difference in distant metastasis-free interval. One study reported that both mastectomy and wide local excision significantly improved local control compared to primary endocrine therapy (Fennessey *et al*, 2004).

One trial did not quantify adverse events, only reporting that one woman from the primary endocrine therapy arm had to drop out of the trial because of endocrine therapy-related adverse effects (Fennessey *et al*, 2004). In another, all patients in the surgery plus tamoxifen arm who had axillary clearance had paraesthesia on the ipsilateral arm and lateral thoracic wall. Tamoxifen-related toxicity was similar between the two arms and included headache, vertigo, itching, hair loss, cystitis, vaginal bleeding, acute thrombophlebitis, nausea, and indigestion (Mustacchi *et al*, 2003). The other two studies did not report adverse events (Willsher *et al*, 1997; Capasso *et al*, 2000). No trial investigated differences in quality of life. The only trial to evaluate differences in psychiatric morbidity (CRC) used the General Health Questionnaire 28 (Goldberg *et al*, 1970), and a socio-demographic questionnaire, which investigated levels of domestic support and social isolation. At 3 months after the start of treatment the surgery group had more psychosocial morbidity (*P*=0.03); however, there was no difference between the surgery and primary endocrine therapy groups at 2 years (Fallowfield, 1994).

## DISCUSSION

### Statement of principal findings

This study has demonstrated that primary endocrine therapy is inferior to surgery with endocrine therapy for the local control of breast cancer in ER-unselected, medically fit older women. This is independent of the type of surgery, with both mastectomy and wide excision (without adjuvant radiotherapy) achieving superior local control. The meta-analysis showed no significant difference in OS between the two treatments in this group of women. One trial showed a small but significant survival advantage for surgery with adjuvant endocrine therapy, where follow-up was extended to 13 years (Fennessey *et al*, 2004). There are no data on formal QoL assessments between these two groups and no data on patients’ preferences.

### Strengths and weaknesses of the study

The results of this review are based on a limited number of small studies of variable methodological quality. In some cases, the internal validity of the primary studies was affected by competing risks and informative censoring, which violate the assumptions underlying the Kaplan–Meier survival analysis method (these issues are discussed fully elsewhere in Hind *et al*, 2006). Heterogeneity between trials, in terms of interventions and outcome assessment, also made assessment of some outcomes problematic. There was considerable variation in surgical technique to the breast and the axilla (Table 1). It was unclear whether surgical margins were adequate by modern standards in any trial. In one trial, case selection for breast conservation included women with large tumours (T3 and T4) which would be inappropriate by modern standards (Gazet *et al*, 1994). Two of the trials which offered women wide local excision (St Georges and CRC) did not report using adjuvant radiotherapy which is standard practice today and may have reduced the clinical effect size of surgery (Gazet *et al*, 1994; Fennessey *et al*, 2004). In the analysis of local disease control in the second comparison (surgery with adjuvant tamoxifen *vs* primary tamoxifen) statistical heterogeneity cannot be explained by differences in population or treatment characteristics and is likely to be an artefact of the CRC trial's longer follow-up time.

Most trials recruited women regardless of oestrogen receptor status. Only 85–90% of women in this age group have ER-positive tumours (Diab *et al*, 2000). For the remainder, tamoxifen is not an active intervention and their treatment with tamoxifen is not therefore in line with modern clinical practice. The inclusion of such women may also have biased the results of the meta-analysis. Had such women been excluded from included trials, the primary endocrine therapy arms may have performed better against surgery arm plus endocrine therapy. However, the one trial to recruit exclusively patients with ER-positive tumours found local control to be superior with surgery and endocrine therapy (Willsher *et al*, 1997). Furthermore, none of the included studies controlled for patient co-morbidity and even among those fit for surgery in this age group, a significant proportion of patients will die of co-morbid diseases so reducing the relative advantages of any breast cancer therapies (Satariano and Ragland, 1994).

### Strengths and weaknesses in relation to other studies

This is the first systematic review on this topic and provides an overview of all primary studies on the subject.

### Meaning of the study possible explanations and implications for clinicians and policymakers

Primary endocrine therapy should only be offered to women with ER-positive tumours who are unfit for, or who refuse, surgery. In a cohort of women with reduced life expectancy, owing to significant co-morbid disease, and ER-positive tumours, primary endocrine therapy may be an appropriate treatment choice. A national UK trial will shortly be starting to evaluate selection criteria for the use of primary endocrine therapy, (Endocrine +/− Surgical Therapy for Elderly women with Mammary cancer, ESTEEM), which will aid in decision making.

### Unanswered questions and future research

Since these studies were designed, endocrine therapies other than tamoxifen have become available. Aromatase inhibitors have been shown to be superior to tamoxifen in the adjuvant setting and may by attractive as primary endocrine therapy for older women who are unfit for surgery. Trials are needed to test this hypothesis. The ESTEEM trial will use aromatase inhibitors rather than tamoxifen, which may enhance the efficacy of primary endocrine therapy.

## Figures and Tables

**Table 1:** Study characteristics

**Study**	**Participants**	**Interventions**	**Outcomes**	**Allocation concealment**
EORTC 10851 (UK) *n*=164 (Fentiman *et al*, 2003)	Women (aged 70+) with operable breast cancer	Surgery (mastectomy; full axillary clearance) *vs* tamoxifen (20 mg day^−1^)	Survival - overall; PFS; local disease control; distant metastasis free survival	A
Nottingham 1 (UK) *n*=131 (Kenny *et al*, 1998)	Women (aged 70+) with operable breast cancer	Surgery (wedge mastectomy; limited axillary surgery) *vs* tamoxifen (40 mg day^−1^)	Survival - overall; PFS; local disease control; distant metastasis free survival	B
St Georges (UK) *n*=200 (Gazet *et al*, 1994)	Women (aged 70+) with operable breast cancer	Surgery (mastectomy or wide local excision without radiotherapy; axillary surgery not specified) *vs* tamoxifen (20 mg day^−1^)	Survival - overall; PFS; local disease control; distant metastasis free survival	B
CRC (UK) *n*=455 (Fennessey *et al*, 2004)	Women (aged 70+) with operable breast cancer	Surgery (mastectomy or wide local excision without radiotherapy; axillary surgery not specified) plus tamoxifen (40 mg day^−1^) *vs* tamoxifen alone	Survival - overall; PFS; local disease control; distant metastasis free survival; psychiatric and social morbidity	A
GRETA (Italy) *n*=274 (Mustacchi *et al*, 2003)	Women (aged 70+) with operable breast cancer	Surgery (mastectomy or wide local excision with radiotherapy; axillary clearance) plus tamoxifen (20 mg day^−1^) *vs* tamoxifen alone	Survival - overall; PFS; local disease control; distant metastasis free survival	A
Naples (Italy) *n*=75 (Capasso *et al*, 2000)	Women (aged 70+) with operable breast cancer	Surgery (mastectomy or wide local excision with radiotherapy;±axillary clearance) plus tamoxifen (20 mg day^−1^) *vs* tamoxifen alone	Survival - overall; PFS	B
Nottingham 2 (UK) *n*=147 (Willsher *et al*, 1997)	Women (aged 70+) with operable breast cancer	Surgery (wedge mastectomy; limited axillary surgery) plus tamoxifen *vs* tamoxifen (20 mg day^−1^)	Survival - overall; PFS	B

PFS=progression-free survival.

Surgery *vs* primary endocrine therapy.

**Table 2:** Surgery *vs* primary endocrine therapy results

**Trial**	**Median follow-up**	**Surgery *n*/*N***	**Primary endocrine therapy *n*/*N***	**HR (95% CI)**
				
Surgery primary endocrine therapy				
*Mortality (‘OS’)*				
EORTC 10851	10 years	60/82	50/82	1.11 (0.75–1.65)
Nottingham 1	5 years	28/65	28/66	1.06 (0.59–1.92)
St Georges	6 years	28/100	33/100	0.75 (0.44–1.26)
				
*Mortality, recurrence or progression (‘PFS’)*				
EORTC 10851	10 years	63/82	69/82	0.55 (0.39–0.77)
Nottingham 1	12 years	56/65	57/66	Not estimable
St Georges	6 years	60/100^a^	70/100^a^	Not estimable
				
*Local recurrence or local progression as first event*				
EORTC 10851	10 years	7/82	47/82	Not calculated^b^
Nottingham 1	9 years	16/65^a^	45/66^a^	Not calculated^b^
St Georges	6 years	36/100	53/100	Not calculated^b^
				
*Distant metastases as first or simultaneous event*				
EORTC 10851	10 years	15/82	7/82	Not calculated^b^
Nottingham 1	12 years	NR	NR	Not calculated^b^
St Georges	6 years	14/100	8/100	Not calculated^b^

OS=overall survival; PFS=progression-free survival.

a Individual patient data from trial list.

b Not calculated because of 20–50% competing risks (Nottingham 1 and EORTC 10851) and informative censoring (St Georges). Surgery plus adjuvant endocrine therapy *vs* primary endocrine therapy.

**Table 3:** Surgery plus adjuvant endocrine therapy *vs* primary endocrine therapy results

**Trial**	**Median Follow up**	**Surgery *n*/*N***	**Primary endocrine therapy *n*/*N***	**HR (95% CI)**
Surgery plus endocrine therapy *vs* primary endocrine therapy				
*Mortality (‘OS’)*				
CRC	13 years	159/225	187/230	0.78 (0.63–0.96)
GRETA	7 years	130/239	144/235	0.98 (0.77–1.25)
Nottingham 2	5 years	8/53	14/94	0.80 (0.73–2.32)
				
*Mortality or progression (‘PFS’)*				
CRC	13 Years	NR	NR	NR
GRETA	7 years	140/239	188/235	0.65 (0.53–0.81)
Nottingham 2	5 years	NR	NR	NR
				
*Local recurrence or local progression as first event*				
CRC	13 years	36/225	115/230	0.25 (0.19–0.32)
GRETA	7 years	27/239	95/235	0.38 (0.25–0.57)
Nottingham 2	3 years	2/53	30/94	Not estimable
				
*Distant metastases as first or simultaneous event*				
CRC	13 years	20/225	14/235	Not estimable
GRETA	7 years	0/225	10/235	Not estimable
Nottingham 2	3 years	NR	NR	Not estimable
